# Endometrial transcriptome profiling of patients with recurrent implantation failure during hormone replacement therapy cycles

**DOI:** 10.3389/fendo.2023.1292723

**Published:** 2024-01-30

**Authors:** Wen-bi Zhang, Jue Li, Qing Li, Xiang Lu, Jun-ling Chen, Lu Li, Hua Chen, Wei Fu, Jiu-cheng Chen, Bing-jie Lu, Han Wu, Xiao-xi Sun

**Affiliations:** ^1^ Shanghai Ji Ai Genetics and In vitro Fertilization and Embryo Transfer (IVF-ET) Institute, Obstetrics and Gynecology Hospital, Fudan University, Shanghai, China; ^2^ Unimed Biotech (Shanghai) Co., Ltd., Shanghai, China; ^3^ Key Laboratory of Female Reproductive Endocrine-Related Diseases, Obstetrics and Gynecology Hospital, Fudan University, Shanghai, China

**Keywords:** endometrial receptivity (ER), window of implantation (WOI) displacement, recurrent implantation failure (RIF), hormone replacement therapy (HRT) cycle, differentially expressed gene (DEG)

## Abstract

**Background:**

The molecular mechanisms underlying window of implantation (WOI) displacement in patients with recurrent implantation failure (RIF) remain unclear. This study aims to explore the transcriptomic signatures of endometrium with normal and displaced WOIs and to identify the causes of endometrial receptivity (ER) abnormalities and WOI displacement in RIF patients.

**Methods:**

In this study, 40 RIF patients were recruited and underwent personalized embryo transfer (pET) guided by the predicted results of endometrial receptivity diagnosis (ERD) model. Transcriptome analysis of endometrium from patients with clinical pregnancies after pET was performed to identify differentially expressed genes (DEGs) associated with WOI displacement. Gene expression data from HRT and natural cycle endometrium were compared to identify specific gene expression patterns of ER-related genes during WOI.

**Results:**

The ERD results indicated that 67.5% of RIF patients (27/40) were non-receptive in the conventional WOI (P+5) of the HRT cycle. The clinical pregnancy rate in RIF patients improved to 65% (26/40) after ERD-guided pET, indicating the effectiveness of transcriptome-based WOI prediction. Among the 26 patients with clinical pregnancy, the gene expression profiles of P+5 endometrium from advanced (n=6), normal (n=10) and delayed (n=10) WOI groups were significantly different from each other. Furthermore, 10 DEGs identified among P+5 endometrium of 3 groups were involved in immunomodulation, transmembrane transport and tissue regeneration, which could accurately classify the endometrium with different WOIs. Additionally, a large number of ER-related genes showed significant correlation and similar gene expression patterns in P+3, P+5, and P+7 endometrium from HRT cycles and LH+5, LH+7, and LH+9 endometrium from natural cycles.

**Conclusion:**

Our study shows that ER-related genes share similar gene expression patterns during WOI in both natural and HRT cycles, and their aberrant expression is associated with WOI displacements. The improvement of pregnancy outcomes in RIF patients by adjusting ET timing according to ERD results demonstrates the importance of transcriptome-based endometrial receptivity assessment and the clinical efficiency of ERD model.

## 1 Introduction

Implantation failure remains an unsolved quandary in reproductive medicine and a severe obstacle in human assisted reproduction treatment (ART) (1). Most *in vitro* fertilization and embryo transfer (IVF-ET) attempts ended in implantation failure with ~30% successful live birth (2–4). Moreover, nearly 10% of patients undergoing IVF-ET suffer from recurrent implantation failure (RIF), which is defined as failing to achieve clinical pregnancy after transfer of 4 or more high-quality embryos in more than three fresh or frozen cycles for a woman (5). Due to illusive etiology, RIF is still a major challenge and leads to tremendous distress and frustration for both patients and clinicians (6).

Successful embryo transfer (ET) requires embryos with development potential and a receptive endometrium, as well as a synchronized molecular dialogue between them (7). Much effort has been put into embryo quality screening, but more than 40% of implant failures with high-quality euploid embryos suggested that endometrium and its receptivity also play a pivotal role in embryo implantation (8). Several studies have shown that endometrial receptivity (ER) defects interfere with ET and are considered to be responsible for the majority of implantation failures (1, 9–13). This means that to achieve successful ET, in addition to embryo information, an accurate diagnostic test of ER status is required before IVF-ET.

Generally, the endometrium will enter a narrow window of receptive state regulated by steroid hormone regulation during the mid-secretory phase for allowing embryo implantation, known as the window of implantation (WOI) (14). In conventional IVF-ET protocols, the transfer timing of frozen-thawed blastocysts (embryos on days 5) is recommended to be the day LH+7 (7th day after LH surge) of a natural cycle or day P+5 (5th day after strating progesterone administration) of a hormone replacement therapy (HRT) cycle (15, 16). However, the evidence suggests that WOI is not uniform across all women due to inter-individual differences in the endometrial genetic constitution, and it can be delayed, advanced, longer or shorter than expected (17–19). Furthermore, a few studies suggest an association between WOI displacement and implant failure in infertile patients undergoing IVF-ET (11, 20). The temporal displacement of WOI has been reported in up to 26% (22/85) and 47% (29/62) of patients with RIF in two studies (12, 21). It has been found that in about 80% of French RIF patients, the receptivity window was delayed in both natural cycles and HRT cycles (22). Combined together, these data highlight the necessity to pinpoint the ER status (receptive or non-receptive) and the WOI of individual patients undergoing IVF-ET, and then adjust the timing for ET according to the personalized WOI (pWOI), particularly in patients with RIF (12, 21, 22).

Both classical methods and new strategies of ER assessment have been widely applied in clinical practice including histological dating, ultrasound endometrial dating, hysteroscopy inspection, or using biomarkers such as osteopontin, integrins, *LIF* and *HOX* genes, etc (23–26). However, it remains challenging to precisely identify the ER status and to further pinpoint WOI, especially in RIF patients (27). Recently, various biomarkers representing the receptive status of the endometrium have been identified by high-throughput assays and their expression dynamics can accurately predict the WOI for guiding personalized embryo transfer (pET) (11, 27). Endometrial receptivity array (ERA), a customized microarray developed based on gene expression profiling, has been commercially applied for WOI prediction and demonstrated to significantly improve pregnancy outcomes in RIF patients through more precise timing for pET than the classical histological dating method (13, 21, 28, 29). Compared with microarray, high-throughput sequencing-based RNA-seq is a more comprehensive and quantitative method for ER gene expression profiling and is completely independent of prior knowledge (30, 31). Nevertheless, all the array-based studies need to be re-evaluated by NGS-based methods due to independent technical limitations (32). Especially, it is necessary to investigate the global endometrial gene expression profiles of RIF patients around WOI based on RNA-seq.

Most previous ER studies profiled endometrial transcriptome controlled ovarian stimulation (COS) and natural cycles just by comparing differences in gene expression profiles between the pre-receptive (early-secretory) and the receptive (mid-secretory) phase, rather than 3 phases including the post-receptive phase (18, 33). Moreover, only a few of these studies compared gene-expression profiles from the same patient (34, 35). It is advisable to exclude individuals with obvious pathology and to sample by endometrial biopsies at different phases of the same cycle in each patient to minimize patient-to-patient and batch-to-batch variance as well as show true differences. In addition, most previous ER studies mainly focused on North American and European populations. Considering that ethnic backgrounds may also lead to significant transcriptome differences, it is necessary for us to launch a study targeting Chinese RIF patients. Additionally, investigating the endometrial transcriptomic signatures of HRT cycles in RIF patients who proved fertile by pET pregnancies is more useful for RIF study than previous endometrial transcriptome studies.

In our previous study, we found significant differences in the endometrial transcriptome across different phases of natural cycle in healthy Chinese women (36). Based on the specific endometrial transcriptome signatures and combined with machine learning algorithms, we developed a transcriptome-based endometrial receptivity diagnostic (ERD) model for WOI prediction, which contains 166 biomarker genes and shows 100% prediction accuracy in the training set. In this study, we applied this ERD model to determine the ER status of RIF patients and to guide pET. Furthermore, we aimed to identify individuals with WOI displacement in RIF patients and to explore the transcriptomic signatures of endometrium with normal and displaced WOI during HRT cycle, which could be helpful to further understand the causes of ER abnormalities and the underlying molecular mechanism of WOI displacement in RIF patients.

## 2 Materials and methods

### 2.1 Ethics approval

This study was approved by the ethical committee of Shanghai Ji Ai Genetics and IVF-ET Institute of Obstetrics and Gynecology hospital affiliated with Fudan University (JIAI E2020-015). All subjects participating in the project signed an informed consent form.

### 2.2 Patient selection

In this study, all the participants who need IVF-ET treatment due to male, tubal or other unexplained infertility factors had been identified as RIF (≥3 attempts at embryo transfer with ≥4 high-quality embryos failing to implant). None of the participants had the following diseases: endometriosis, endometritis, hysteromyoma, adenomyosis, endometrial hyperplasia, thin endometrium (<7mm), intrauterine adhesion, endometrial polyps, hydrosalpinx, reproductive-tract malformation (e.g., septate uteri), polycystic ovarian syndrome, thyroid dysfunction, hyperprolactinemia, immunological or thrombotic disorders. None of them had taken any aspirin within 3 months.

Totally, 40 RIF patients (with a mean of 4.55 ± 2.28 prior failures) aged between 25-39 with a body mass index (BMI) of 18-27 kg/m^2^ were recruited for ERD testing with P+5 endometrial samples, followed by pET based on ERD results. The high-quality embryos used for pET are day-5 blastocysts of at least 4BB according to Gardner’s classification (37). Clinical pregnancy was ranked as successful ERD-guided pET, which was defined as ultrasonographic evidence of an intrauterine sac with heartbeat at the 6th gestational week (38). The patients who had undergone pET without clinical pregnancy were defined as patients with “uncertain WOI”. Finally, only 26 patients who achieved clinical pregnancy by pET were selected for further analysis and grouped according to the ET timing (advanced, normal or delayed WOI). Additionally, transcriptome data from the LH+5 (n=20), LH+7 (n=19) and LH+9 (n=25) endometrial samples of natural cycle were obtained from healthy fertile Chinese women recruited in our previous study (36).

### 2.3 Endometrial sampling and sequencing

The endometrial samples were processed and collected using the same protocol as described before (38). Estradiol valerate (Progynova; Bayer, Leverkusen, Germany) was administered at 4~8 mg daily from 2nd day of the menstrual cycle until endometrial thickness was ≥7 mm. On that same day, serum progesterone level was measured. If the progesterone level was ≤1.5 ng/ml, 90 mg progesterone sustained-release vaginal gel (Crinone; Merck-Serono, Darmstadt, Germany) would be applied transvaginally per day. The day of first Crinone use was referred to as P+0. Endometrial biopsies were taken on days P+3, P+5, and P+7 of the same HRT cycle in each patient by one dedicated physician using a disposable uterine-cavity tissue suction tube (Yudu medical apparatus and instruments Co., Ltd., Suzhou City, China). The RNA extraction and sequencing protocols were consistent with our previous study (36). The total RNA was extracted from 10–20 mg of endometrial tissue with an RNAprep Pure Tissue Kit (DP341; TIANGEN Biotech Co., Ltd., Shanghai, China) according to the manufacturer’s instructions. RNA quality was assessed by 2100 Bioanalyzer with an RNA 6000 Pico Kit (Agilent Technologies). Only the RNAs with an RNA Integrity Number (RIN) more than 8.0 were used for further processing. For the sequencing libraries, 1 mg of total RNA was used for poly (A) messenger RNA (mRNA) enrichment by VAHTS mRNA Capture Beads (N401-02; Vazyme). The poly(A) mRNA was then used to generate the sequencing libraries using a KAPA Stranded RNA-Seq Library Preparation Kit (KR0934; Kapa Biosystems Inc., Wilmington, MA, USA) following the manufacturer’s instructions. Finally, the complementary DNA (cDNA) libraries were sequenced by paired-end 150-base pair (bp) on an Illumina Nova-Seq 6000 System (Illumina, Inc., San Diego, CA, USA). Each sample was sequenced to generate approximately 8 Gb of raw data for data analysis.

### 2.4 Data analysis

Transcriptomic data of 73 endometrial samples taken from 26 RIF patients in this study have been uploaded to Gene Expression Omnibus (GEO) database.

The raw sequencing data was processed by fastp with the following criteria: the average quality score of each read >15, read length >75 and the number of “N” bases <5. We removed all reads mapping to ribosomal RNA by SortMeRNA and mapped the remaining reads to the human genome hg19 by STAR with default parameters. Gene expression was calculated by the corresponding transcripts per million (TPM) based on GENCODE v19 annotations (39–43). The group-level differential expression analysis was performed by using the R package edgeR (Bioconductor, https://bioconductor.org/packages/release/bioc/html/edgeR.html). The genes with false discovery rate (FDR)<0.05 and |fold change (FC)|≥2.0 were selected for DEGs analysis.

### 2.5 Statistical analysis

Patient information was shown as mean ± standard error of the mean. Differences between groups were compared by One-way ANOVA test and the proportion of the reasons for IVF-ET among the three groups were compared by chi-square test (df=4), and *p*-value <0.05 was considered to be statistically significant.

### 2.6 Hierarchical clustering

The hierarchical clustering analysis was performed on all paired samples by comparing DEGs (the combined gene list) with the smooth correlation for distance measure algorithm (Gene-Spring) to identify samples with similar gene expression patterns. Heat maps were plotted based on the measured intensity values of DEGs with hierarchical tree to indicate the relationships among different groups.

### 2.7 Gene ontology and KEGG pathway analysis

Only genes with significant differential expression (FDR<0.05) were retrieved for gene ontology (GO) and Kyoto Encyclopedia of Genes and Genomes (KEGG) pathway analysis. The GO and KEGG pathway analyses were performed with the R package clusterProfiler (Bioconductor, https://bioconductor.org/packages/release/bioc/html/clusterProfiler.html). The adjusted *P <*0.05 by Benjamini-Hochberg correction was considered statistically significant.

### 2.8 Principal component analysis

Principal component analysis (PCA) was performed on the P+3, P+5 and P+7 samples from the patients with normal WOI to visualize the differences between samples. The PCA analysis was done by RAGE.

### 2.9 Pearson’s correlation analysis

Pearson’s correlation analysis was used to calculate the correlation of endometrial gene expression between natural and HRT cycles. Correlation coefficients r>0.3 and *P*<0.001 for both data sets were considered significantly correlated.

### 2.10 Mfuzz clustering analysis

Mfuzz clustering analysis was performed to classify the genes with similar expression patterns in natural and HRT cycles by applying the fuzzy c-means algorithm of the Mfuzz R package (https://www.bioconductor.org/packages/release/bioc/html/Mfuzz.html). The average TPM value for each gene at each phase of natural or HRT cycles was used as the input. Each gene was assigned a unique cluster based on its membership value after standardization (standard deviation of less than 1 would be filtered).

## 3 Results

### 3.1 Patient information

**Figure 1:**
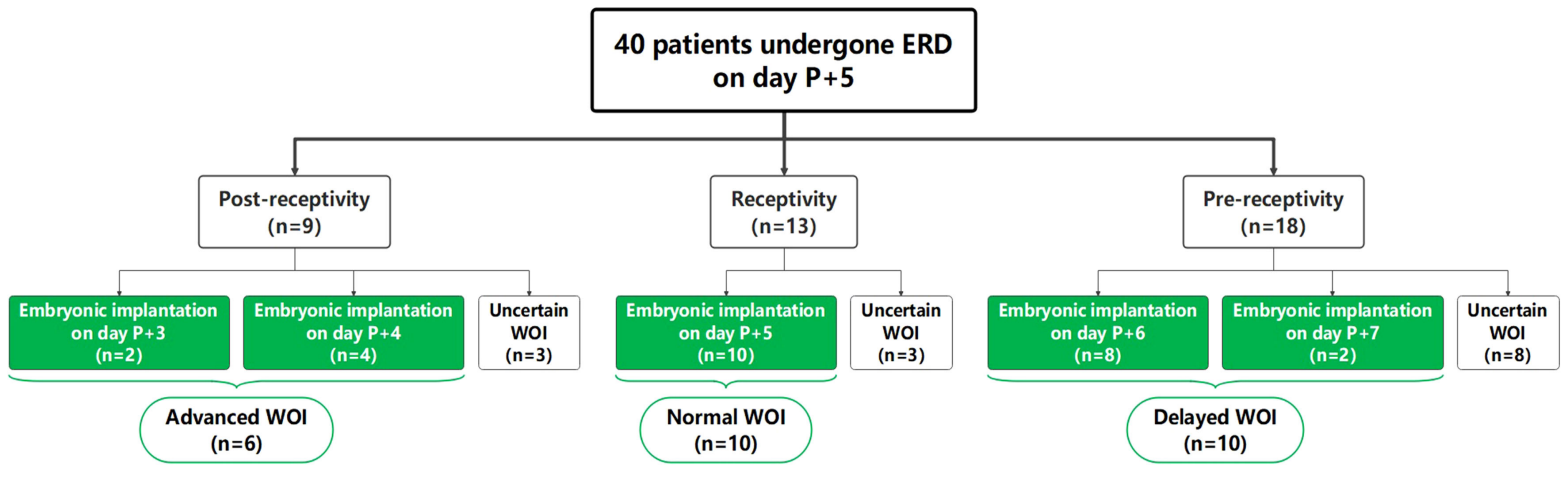
Summary of ERD results and distribution of recruited RIF patients. “Uncertain WOI” refers to patients who had not yet undergone embryo transfer, or who had undergone embryo transfer without clinical pregnancy. The patients marked in green underwent successful ERD-guided pET and achieved clinical pregnancy.

Overall, 40 RIF patients were recruited for this study and pET was performed based on ERD results of their P+5 samples. As shown in **Figure 1**, the ERD results of P+5 samples indicated a receptive profile of 32.5% (13/40) and a non-receptive profile of 67.5% (27/40). Of the non-receptive samples, 66.7% (18/27) were classified as pre-receptive state and 33.3% (9/27) as post-receptive state.

Among the 40 patients, 26 patients achieved successful clinical pregnancy and were selected for further analysis. The other 14 patients were classified as “uncertain WOI” (see Materials and Methods). According to the ET timing, 26 patients were further divided into the advanced WOI group (receptive on P+3 and P+4, n=6), normal WOI group (receptive on P+5, n=10) and delayed WOI group (receptive on P+6 and P+7, n=10), respectively. There was no significant difference in age, BMI, number of prior implantation failures and transferred embryos, cycle length, basal serum follicle stimulating hormone (FSH) level, progesterone level on the day of starting progesterone administration and endometrium thickness at the time of biopsy among the three groups of RIF patients (**Table 1**).

**Table 1:** Demographic characteristics of the 26 RIF patients in different groups.

Characteristic	Advanced Group	Normal Group	Delayed Group	*p*-value
Patients (N)	6	10	10	
Age (years): Mean±SD	31.67 ± 4.80	31.40 ± 4.09	32.10 ± 3.67	0.9291
BMI ^a^ (kg/m^2^): Mean±SD	20.45 ± 1.35	21.30 ± 2.15	22.29 ± 2.70	0.2995
Length of the menstrual cycle (days): Mean±SD	32.50 ± 4.18	31.11 ± 3.16	29.83 ± 2.03	0.3722
Prior implantation failures (N): Mean±SD	4.50 ± 2.81	4.70 ± 2.26	4.40 ± 2.41	0.9623
Prior transferred embryos (N): Mean±SD	6.17 ± 1.46	6.10 ± 1.97	6.60 ± 1.85	0.8302
Embryos transferred (N): Mean±SD	1.33 ± 0.27	1.3 ± 0.23	1.5 ± 0.28	0.6572
Serum hormone levels:				
FSH ^b^ (mIU/ml): Mean±SD	8.25 ± 1.63	7.04 ± 1.03	8.05 ± 1.98	0.2927
Pg ^c^ (ng/ml): Mean±SD	0.34 ± 0.17	0.37 ± 0.19	0.39 ± 0.17	0.8536
Endometrium thickness (mm): Mean±SD	9.17 ± 1.67	8.80 ± 1.25	8.70 ± 1.27	0.8188
Reasons for IVF-ET:				
Male factor (N)	2	3	2	
Tubal factor (N)	4	6	5	
Unexplained factor (N)	0	1	3	
Collected endometrial samples:				
P+3 samples (N)	6	10	9	
P+5 samples (N)	6	10	10	
P+7 samples (N)	4	9	9	

a BMI, Body Mass Index. The body mass index is the weight in kilograms divided by the square of the height in meters.

b FSH, Follicle stimulating hormone. Basal serum FSH level is measured on the third day of the last menstruation for each participant.

c Pg, progesterone. Serum progesterone level is measured on the day of starting progesterone management.

### 3.2 Endometrial gene expression profiles of HRT cycle in RIF patients

**Figure 2:**
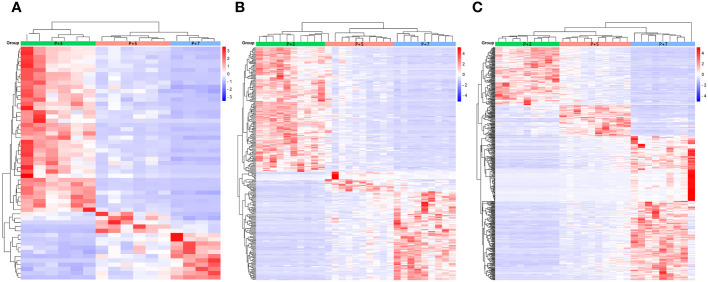
Hierarchical clustering of the phase-grouped DEGs and endometrial samples from RIF patients with advanced WOI **(A)**, normal WOI **(B)**, and delayed WOI **(C)**, respectively. Corresponding time points of sample collection were indicated by color-coded bars on top of the heat map: P+3 (green), P+5 (pink), and P+7 (blue). In the heat map, each row represents a single gene and each column represents a single sample. Color indicates the intensity of the gene expression value (red=high; blue=low), as shown in the color legend on the right.

To systematically profile the endometrial transcriptome of RIF patients with different WOIs, unsupervised hierarchical clustering was used to cluster the endometrial samples collected from different phases of the HRT cycle in three groups. Within each WOI group, samples from different phases of HRT cycle showed significant differences in gene expression patterns and can be clustered into three well-defined branches (**Figure 2**). In both the advanced and normal WOI groups, the P+5 and P+7 samples were classified into the same sub-tree, suggesting similar gene expression profile for receptive and post-receptive endometrium (**Figures 2A, B**). While in the delayed WOI group (**Figure 2C**), the P+3 and P+5 samples were clustered into one sub-tree, implying pre-receptive status for both phases and the WOI would be delayed. In addition, we identified DEG intersections among the three comparisons (P+3 vs. P+5, P+5 vs. P+7 and P+3 vs. P+7) in advanced (55 DEGs), normal (247 DEGs) and delayed (494 DEGs) WOI groups, respectively (|FC|≥2.0, FDR<0.05; **Supplementary Table 1**). However, the DEG composition of the three WOI groups was dramatically different with only three shared DEGs (*TCN1*, *TUBA4A*, *SULT1E1*), suggesting that diverse WOIs correspond to different endometrial gene expression profiles in the HRT cycle.

### 3.3 Endometrial transcriptomic signatures of RIF patients at the conventional WOI (days P+5) of HRT cycles

**Figure 3:**
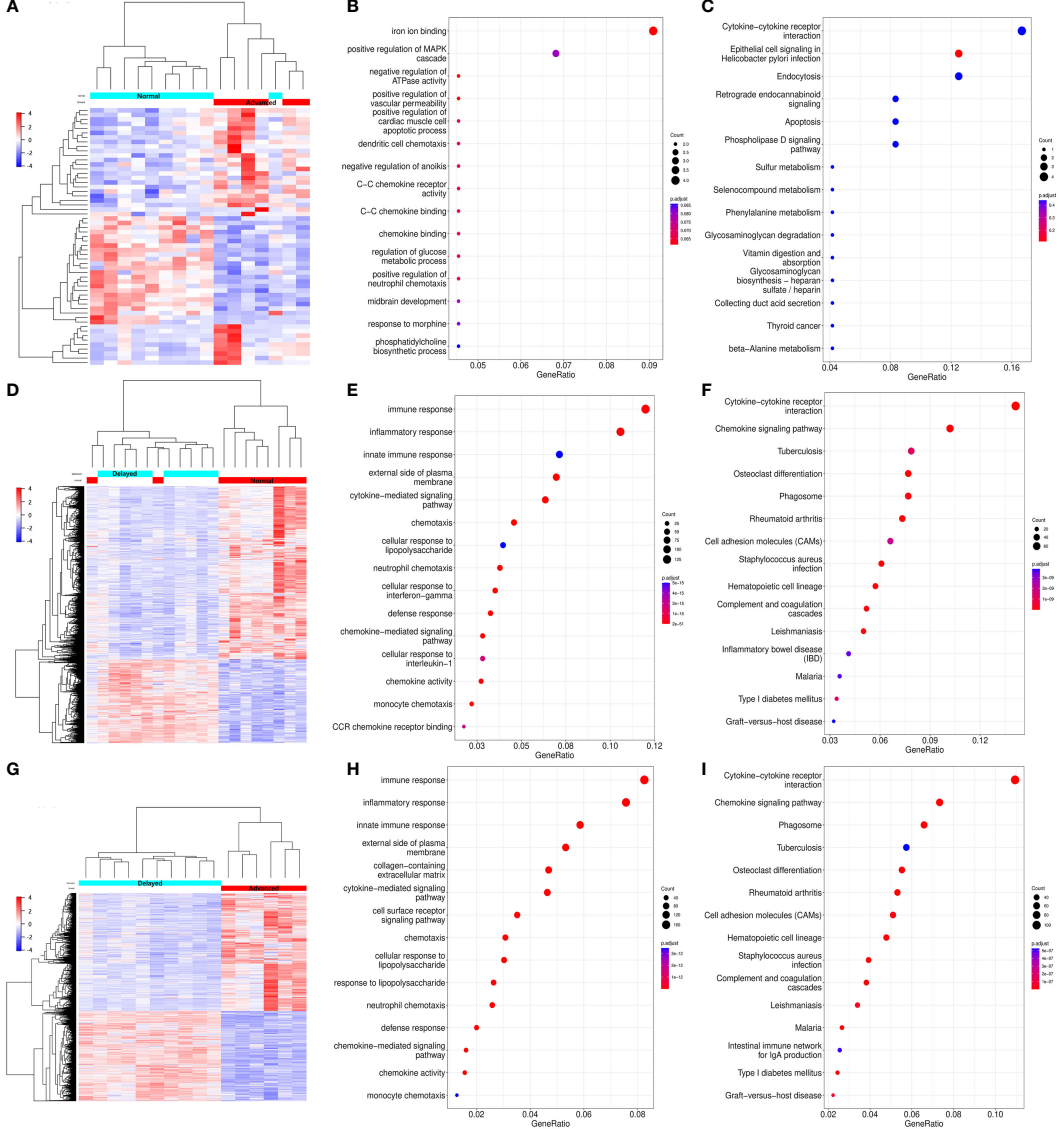
Differential expression and functional enrichment analyses of the DEGs from the P+5 endometrial samples with different WOIs. Hierarchical clustering of the DEGs and samples in comparing Advanced vs. Normal **(A)**, Delayed vs. Normal **(D)**, and Advanced vs. Delayed **(G)** groups. Top 15 GO terms and top 15 KEGG pathways of DEGs in comparing Advanced vs. Normal **(B, C)**, Delayed vs. Normal **(E, F)**, and Advanced vs. Delayed **(H, I)** groups. In the heat map, each row represents a single gene and each column represents a single sample. Color indicates the intensity of the gene expression value (red=high; blue=low), as shown in color legend on the right. The sizes of dots in the GO and KEGG dot-plots indicate gene counts in the corresponding terms and pathway, while the colors of dots indicate the adjusted *P*-value.

To investigate the endometrial transcriptome of RIF patients with WOI displacement, DEGs were identified by comparing the P+5 endometrial samples from three groups. Endometrium with different WOIs could be accurately classified into separate groups by hierarchical clustering based on DEGs (**Figures 3A, D, G**). Compared with the normal WOI group, there were 33 genes significantly up-regulated and 24 genes down-regulated in the advanced WOI group; 435 genes significantly up-regulated and 902 genes down-regulated in the delayed WOI group (FDR<0.05; **Supplementary Table 2**). Additionally, we identified 1,464 up-regulated and 1,116 down-regulated genes by comparing advanced vs. delayed groups, suggesting a strong difference in gene expression between the two groups.

GO and KEGG analysis revealed that the genes differentially expressed between the P+5 endometrium from advanced and normal WOI groups were mainly involved in ion binding, chemotaxis, metabolism and apoptosis (**Figures 3B, C**). While, the DEGs identified by comparing the P+5 endometrium from delayed and normal WOI groups were mainly involved in immunomodulation, signaling, proliferation and cell adhesion (**Figures 3E, F**). In addition, GO and KEGG analysis results of advanced vs. delayed groups showed similar results to those of delayed vs. normal groups (**Figures 3H, I**). These data suggest that patients with different WOIs have distinct gene expression patterns at days P+5 and DEGs can discriminate patients with different WOIs.

### 3.4 Differentially expressed genes associated with WOI displacement

**Figure 4:**
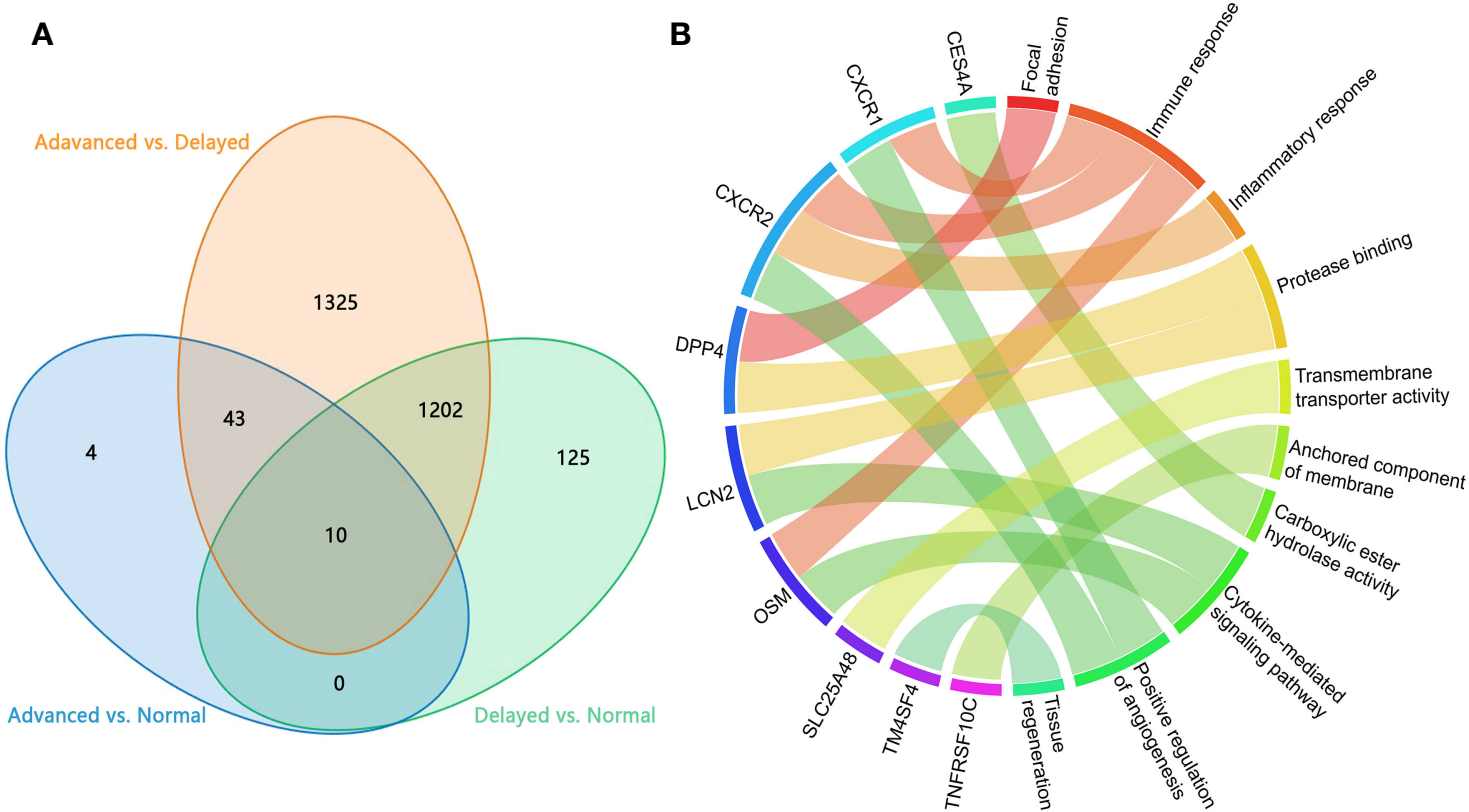
Analysis of the DEGs in P+5 endometrium with normal and displaced WOIs. **(A)** Venn diagram of the DEGs identified in three pairwise comparisons. **(B)** GO terms enriched among the genes associated with WOI displacement. Genes are presented on the left side and the correlating GO terms are presented on the right side of the chord.

**Figure 5:**
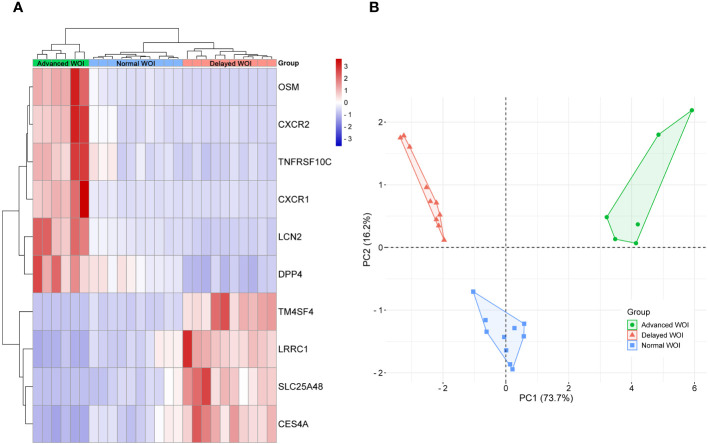
Unsupervised classification of P+5 endometrial samples base on the expression data of the 10 DEGs associated with WOI displacement. **(A)** Unsupervised hierarchical clustering was performed base on the gene expression data of 10 DEGs to separate the P+5 endometrial samples into advanced WOI (green), normal WOI (blue), or delayed (red) WOI groups. In the heat map, each row represents a single gene and each column represents a single sample. The relative expression of each gene is color-coded as high (red) or low (blue) (color legend on the right of the heat map). **(B)** Principal component analysis (PCA) was performed base on the gene expression data of 10 DEGs to separate the P+5 endometrial samples and confirmed the unsupervised cluster analysis. Green dots denote P+5 samples with advanced WOI (n=6), red triangles denote P+5 samples with delayed WOI (n=10) and blue squares denote P+5 samples with normal WOI (n=10).

To identify critical genes associated with WOI displacement, we intersected DEGs among the three pairwise comparisons (advanced vs. normal, advanced vs. delayed and delayed vs. normal) (**Figure 4A**). A total of 10 DEGs were shared among all three comparisons (FDR<0.05; **Table 2**). Compared to the normal WOI group, 6 out of the 10 genes were significantly up-regulated in the advanced WOI group on days P+5, including *DPP4*, *CXCR1*, *CXCR2*, *OSM*, *LCN2* and *TNFRSF10C*. While the remaining 4 genes, including *TM4SF4*, *LRRC1*, *SLC25A4*8 and *CES4A*, were significantly up-regulated in the P+5 endometrium of delayed WOI group. Surprisingly, both hierarchical clustering and PCA showed that the expression levels of these 10 genes can classify the P+5 samples of RIF patients with different WOIs (**Figures 5A, B**). Furthermore, GO analysis of the 10 DEGs showed that these genes were mainly related to immunomodulation, transmembrane transport and tissue regeneration (**Figure 4B**). Combined together, these data suggest that these 10 genes may play important roles in WOI displacement and may be novel additions to the list of biomarkers for predicting WOI (See Discussion).

**Table 2:** Ten genes significantly differentially expressed among the P+5 samples from advanced, normal and delayed WOI groups.

Number	Gene ID	Gene symbol	Gene name	Advanced vs. Normal		Delayed vs. Normal		Advanced vs. Delayed	
				FC ^a^	FDR ^b^	FC	FDR	FC	FDR
1	ENSG00000197635	*DPP4*	Dipeptidyl peptidase IV	2.28	0.016940245	-2.17	0.000104966	4.90	1.43257E-23
2	ENSG00000163464	*CXCR1*	C-X-C motif chemokine receptor 1	12.61	0.001678979	-3.61	0.004644961	45.02	1.27941E-11
3	ENSG00000180871	*CXCR2*	C-X-C motif chemokine receptor 2	6.76	0.016940245	-3.62	0.003613223	24.25	2.70453E-12
4	ENSG00000148346	*LCN2*	Lipocalin 2	5.04	0.009457222	-2.69	0.001107467	13.41	1.65567E-15
5	ENSG00000099985	*OSM*	Oncostatin M	10.97	0.016940245	-7.00	1.08401E-07	76.34	3.60392E-12
6	ENSG00000173535	*TNFRSF10C*	TNF receptor superfamily member 10c	3.84	0.038351325	-2.00	0.007396199	7.63	5.7918E-09
7	ENSG00000169903	*TM4SF4*	Transmembrane 4 L six family member 4	-14.33	0.007953374	9.06	0.000571073	-130.68	3.07566E-09
8	ENSG00000137269	*LRRC1*	Leucine rich repeat containing 1	-2.35	0.016940245	2.03	0.000395602	-4.81	4.46243E-23
9	ENSG00000145832	*SLC25A48*	Solute carrier family 25 member 48	-17.62	0.036771634	4.16	0.018112394	-74.28	6.91207E-40
10	ENSG00000172824	*CES4A*	Carboxylesterase 4A	-2.75	0.015002787	2.04	0.000493127	-5.66	2.16765E-40

a FC, Fold change.

b FDR, False discovery rate.

### 3.5 Correlation of endometrial gene expression patterns between natural and HRT cycles

**Figure 6:**
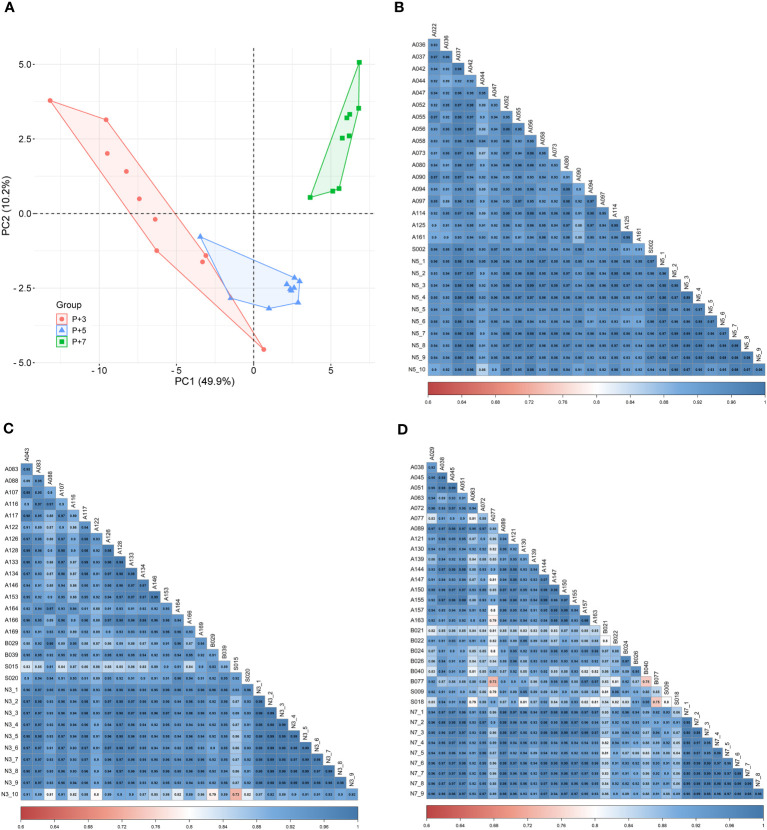
Consistency of endometrial gene expression patterns between natural and HRT cycles. **(A)** Principal component analysis of P+3, P+5, and P+7 samples from the same patients with normal WOI. Red dots denote P+3 samples (n=10), blue triangles denote P+5 samples (n=10) and green squares denote P+7 samples (n=9). Pearson’s correlation analysis of the gene expression data between natural and HRT cycles: **(B)** P+5 samples (n=10) versus LH+7 samples (n=19), **(C)** P+3 samples (n=10) versus LH+5 samples (n=20), **(D)** P+7 samples (n=9) versus LH+9 samples (n=25).

To investigate the correlation of endometrial gene expression patterns between the natural and HRT cycles, we compared gene expression data from the corresponding periods in both cycles and attempted to group endometrium of HRT cycles using transcriptomic signatures found in natural cycle. Interestingly, the PCA result showed that endometrial samples from RIF patients with normal WOI can be phase-grouped according to the 68 DEGs previously identified during the natural cycles (**Figure 6A**) (36). Comparison of the gene expression data between both cycles revealed that 19 of 68 DEGs (|FC|≥2.0, FDR<0.05) were also differentially expressed among the different phases of HRT cycles (P+3, P+5 and P+7) and showed similar gene expression patterns in both cycles. While the remaining 49 genes were not significantly differentially expressed among the 3 phases of HRT cycles, 40 of these genes also showed similar expression patterns in both cycles (**Table 3**). These results indicated that there are common features of gene expression between natural and HRT cycles.

**Table 3:** Expression Patterns of 68 phase-grouped DEGs in natural and HRT cycles.

Gene ID	Gene symbol	Expression patterns	
		Narural cycle	HRT cycle
**ENSG00000066032**	***CTNNA2***	↓ ^a^	↓
**ENSG00000078114**	***NEBL***	↓	↓
**ENSG00000082556**	***OPRK1***	↓	↓
**ENSG00000095932**	***C19orf77***	↓	↓
**ENSG00000099960**	***SLC7A4***	↓	↓
**ENSG00000116983**	***HPCAL4***	↓	↓
**ENSG00000130054**	***FAM155B***	↓	↓
**ENSG00000134020**	***PEBP4***	↓	↓
**ENSG00000140254**	***DUOXA1***	↓	↓
**ENSG00000151150**	***ANK3***	↓	↓
**ENSG00000152931**	***PART1***	↓	↓
**ENSG00000164048**	***ZNF589***	↓	↓
**ENSG00000164120**	***HPGD***	↓	↓
**ENSG00000205795**	***CYS1***	↑ ^b^	↑
**ENSG00000214999**	***AC129492.6***	↓	↓
**ENSG00000237499**	***RP11-356I2.4***	↓	↓
**ENSG00000241106**	***HLA-DOB***	↓	↓
**ENSG00000260186**	***RP11-481J2.2***	↓	↓
**ENSG00000150672**	***DLG2***	↓	↓
ENSG00000019169	*MARCO*	Over-expressed ^c^	Over-expressed
ENSG00000023445	*BIRC3*	↓	↓
ENSG00000105369	*CD79A*	↑	↑
ENSG00000106541	*AGR2*	↑	↑
ENSG00000110347	*MMP12*	Over-expressed	Over-expressed
ENSG00000113763	*UNC5A*	↓	↓
ENSG00000122733	*KIAA1045*	↓	↓
ENSG00000123700	*KCNJ2*	↓	↓
ENSG00000124107	*SLPI*	↑	↑
ENSG00000128510	*CPA4*	↑	↑
ENSG00000132465	*IGJ*	↑	↑
ENSG00000134917	*ADAMTS8*	↓	↓
ENSG00000138109	*CYP2C9*	↑	↑
ENSG00000139514	*SLC7A1*	↓	↓
ENSG00000140090	*SLC24A4*	↓	↓
ENSG00000140479	*PCSK6*	↓	↓
ENSG00000147509	*RGS20*	↑	↑
ENSG00000150594	*ADRA2A*	↑	↑
ENSG00000152315	*KCNK13*	↓	↓
ENSG00000156738	*MS4A1*	↑	↑
ENSG00000157399	*ARSE*	↑	↑
ENSG00000158089	*GALNT14*	↑	↑
ENSG00000158125	*XDH*	↓	↓
ENSG00000165188	*RNF183*	↓	↓
ENSG00000168907	*PLA2G4F*	↓	↓
ENSG00000171056	*SOX7*	↓	↓
ENSG00000172005	*MAL*	↑	↑
ENSG00000172137	*CALB2*	↓	↓
ENSG00000176092	*AIM1L*	↓	↓
ENSG00000179846	*NKPD1*	↓	↓
ENSG00000188064	*WNT7B*	↑	↑
ENSG00000198074	*AKR1B10*	↑	↑
ENSG00000205622	*AF064858.6*	↑	↑
ENSG00000211671	*IGLV2-8*	↑	↑
ENSG00000211899	*IGHM*	↑	↑
ENSG00000212993	*POU5F1B*	↓	↓
ENSG00000225329	*RP11-325F22.5*	↑	↑
ENSG00000226337	*RP11-274B18.4*	↑	↑
ENSG00000241351	*IGKV3-11*	↑	↑
ENSG00000253978	*CTB-178M22.2*	↓	↓
ENSG00000101342	*TLDC2*	↑	Under-expressed ^d^
ENSG00000122641	*INHBA*	Over-expressed	↑
ENSG00000149968	*MMP3*	Over-expressed	↑
ENSG00000172061	*LRRC15*	Under-expressed	↑
ENSG00000185988	*PLK5*	Under-expressed	↓
ENSG00000196611	*MMP1*	Over-expressed	↑
ENSG00000211893	*IGHG2*	↑	Over-expressed
ENSG00000223392	*CLDN10-AS1*	↑	Under-expressed
ENSG00000239951	*IGKV3-20*	↑	Over-expressed

N.B. 19 genes significantly differentially expressed among days P+3, P+5, and P+7 of the HRT cycle are highlighted in bold.

a "↓" indicates that genes were down-regulated during days LH+5 to LH+9 of the natural cycle or days P+3 to P+7 of the HRT cycle.

b "↑" indicates that genes were up-regulated during days LH+5 to LH+9 of the natural cycle or days P+3 to P+7 of the HRT cycle.

c "Over-expressed" indicates that genes were over-expressed on days LH+7 of the natural cycle or days P+5 of the HRT cycle.

d "Under-expressed" indicates that genes were under-expressed on days LH+7 of the natural cycle or days P+5 of the HRT cycle.

To further determine the concordance of endometrial gene expression between natural and HRT cycles, we performed a correlation analysis of gene expression between the LH+7 samples from natural cycles (n=19) and P+5 samples from the normal WOI group (n=10). A significant correlation was observed between any sample from natural and HRT cycles (Pearson’s r>0.85, *P*<0.0001) (**Figure 6B**). Similarly, the correlation of the gene expression between natural and HRT cycles also extended to other phases of the cycle: pre-receptivity phase LH+5 samples (n=20) versus P+3 samples (n=10) (Pearson’s r>0.72, *P*<0.0001) and post-receptivity phase LH+9 samples (n=25) versus P+7 samples (n=9) (Pearson’s r>0.73, *P*<0.0001) (**Figures 6C, D**).

**Figure 7:**
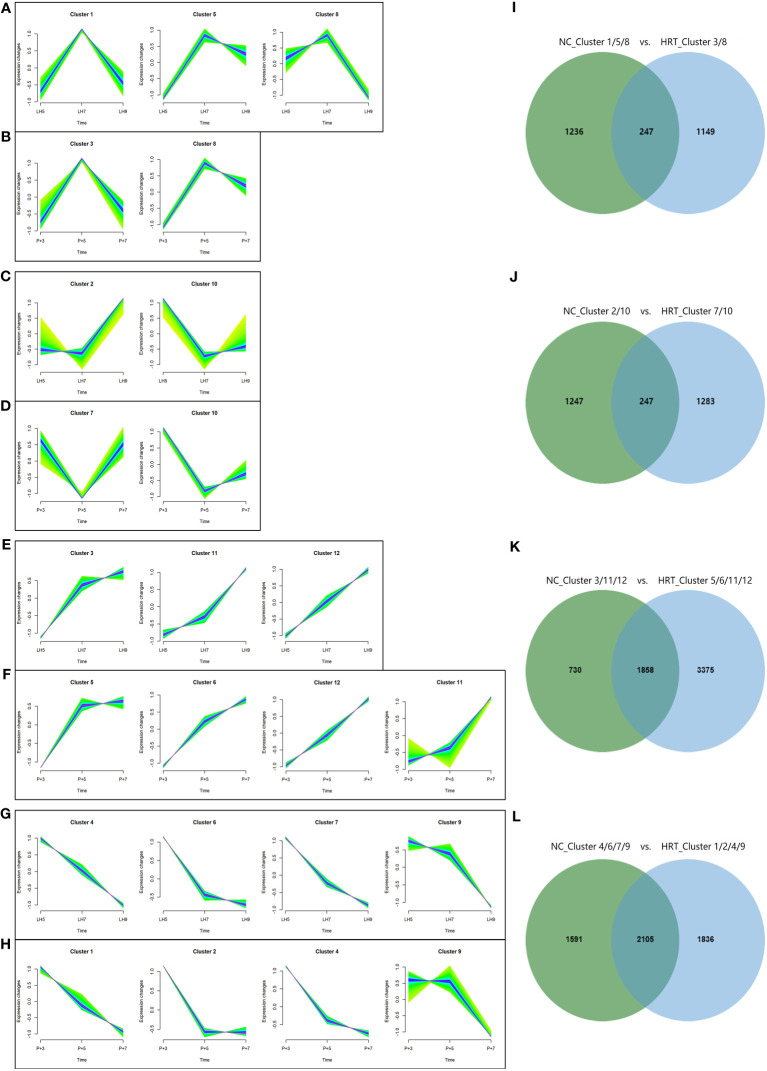
Clustering analysis of genes with similar expression patterns in natural and HRT cycles. The 9,261 genes of natural cycle and 12,100 genes of HRT cycle were respectively grouped into 12 clusters and were further classified into four categories based on the similarity of expression patterns: over-expression **(A, B)** and under-expression **(C, D)** during the conventional WOI (days LH+7 or P+5), continuously up-regulated **(E, F)** and down-regulated expression **(G, H)** during days LH+5 to LH+9 of the natural cycle or days P+3 to P+5 of the HRT cycle. Venn diagrams **(I–L)** show the number of genes classified in four categories of the natural and HRT cycles.

To further compare gene expression dynamics with different patterns in natural and HRT cycles, soft clustering analysis was used to assign genes with similar expression patterns. The 9,261 genes of the natural cycle and 12,100 genes of the HRT cycle were classified into 12 clusters, respectively (**Supplementary Table 3**). These gene clusters were further classified into four categories based on the similarity of expression dynamics: over-expression (**Figures 7A, B**) and under-expression (**Figures 7C, D**) at the conventional WOI (days LH+7 or P+5), continuously up-regulated (**Figures 7E, F**) and down-regulated expression (**Figures 7G, H**) from days LH+5 to LH+9 of the natural cycle or days P+3 to P+7 of the HRT cycle. Within each of the 4 categories, both natural and HRT cycles shared 247, 247, 1858 and 2105 genes (**Figures 7I–L**), respectively. Consequently, we found that numerous genes of endometrium follow similar temporal regulation patterns around the WOI in both natural and HRT cycles, including 10 DEGs mentioned above and the genes identified as putative ER biomarkers in previous transcriptome studies (**Table 4**) (28, 33, 44, 45).

**Table 4:** Expression patterns of 30 ER-related genes in natural and HRT cycles.

Gene ID	Gene symbol	Expression patterns		Reference
		Narural cycle	HRT cycle	
ENSG00000122133	*PAEP*	↑ ^a^	↑	(28, 33, 44, 45)
ENSG00000211445	*GPX3*	↑	↑	(28, 33, 44, 45)
ENSG00000189221	*MAOA*	↑	↑	(28, 33, 44, 45)
ENSG00000118785	*SPP1*	↑	↑	(28, 33, 44, 45)
ENSG00000163993	*S100P*	Over-expressed ^b^	Over-expressed	(28, 33, 44, 45)
ENSG00000196975	*ANXA4*	Over-expressed	↑	(28, 33, 44, 45)
ENSG00000172201	*ID4*	↑	↑	(28, 33, 44, 45)
ENSG00000107984	*DKK1*	↑	↑	(28, 33, 44, 45)
ENSG00000123838	*C4BPA*	↑	Over-expressed	(28, 33, 44, 45)
ENSG00000149131	*SERPING1*	↑	↑	(28, 33, 44, 45)
ENSG00000164136	*IL15*	↑	↑	(28, 33, 44, 45)
ENSG00000169429	*IL8*	Over-expressed	Over-expressed	(45)
ENSG00000128342	*LIF*	↑	↑	(28, 44, 45)
ENSG00000145824	*CXCL14*	↑	↑	(28, 44, 45)
ENSG00000196352	*CD55*	↑	↑	(33, 44, 45)
ENSG00000116717	*GADD45A*	↑	↑	(33, 44, 45)
ENSG00000137673	*MMP7*	↑	↑	(33, 44)
ENSG00000185499	*MUC1*	↓ ^c^	↓	(44, 45)
ENSG00000151150	*ANK3*	↓	↓	(28)
ENSG00000163406	*SLC15A2*	↓	↓	(28)
**ENSG00000197635**	***DPP4***	↑	↑	(28, 33)
**ENSG00000163464**	***CXCR1***	↑	↑	
**ENSG00000180871**	***CXCR2***	↑	↑	
**ENSG00000148346**	***LCN2***	Over-expressed	↑	
**ENSG00000099985**	***OSM***	Over-expressed	↑	
**ENSG00000173535**	***TNFRSF10C***	↑	↑	
**ENSG00000169903**	***TM4SF4***	↓	↓	
**ENSG00000137269**	***LRRC1***	↓	↓	(28)
**ENSG00000145832**	***SLC25A48***	↓	↓	
**ENSG00000172824**	***CES4A***	↓	↓	

N.B. The 10 genes associated with WOI displacement were highlighted in bold.

a "↑" indicates that genes were up-regulated during days LH+5 to LH+9 of the natural cycle or days P+3 to P+7 of the HRT cycle.

b "Over-expressed" indicates that genes were over-expressed on days LH+7 of the natural cycle or days P+5 of the HRT cycle.

c "↓" indicates that genes were down-regulated during days LH+5 to LH+9 of the natural cycle or days P+3 to P+7 of the HRT cycle.

In summary, these results strongly suggested that gene expression dynamics around WOI in both natural and HRT cycles are similar, and common gene expression contexts are required for ER in both cycles.

## 4 Discussion

Altered expression of endometrial receptivity-related genes has been proposed as a potential cause of infertility and RIF (7, 12, 15). Investigating the transcriptomic profiles of endometrium with normal and displaced WOI in RIF patients during the HRT cycle is necessary to understand the mechanisms of ER abnormalities, WOI displacement and RIF pathogenesis, as well as to improve the clinical efficacy of IVF-ET.

As previous studies have concluded, most implantation failures in RIF patients were caused by asynchronous development of the embryo and endometrium, i.e., the endometrium was not in optimally receptive state when the embryo was ready for implantation (19, 20). In current study, we found that 67.5% (27/40) of the Chinese RIF patients presented with WOI displacement, i.e., the endometrium was in a non-receptive state during conventional WOI (P+5). And 66.7% (18/27) of the non-receptive patients were pre-receptive. This is consistent with the earlier studies of RIF that the majority (80%) of the WOI displacements in RIF patients are delayed 1 or 2 days (pWOI delayed to days P+6 or P+7) (46). To separate RIF patients with normal or displaced WOI, we took advantage of a transcriptome-based WOI prediction method (ERD) and performed pET for each RIF patient based on prediction results from P+5 endometrium samples. The 26 RIF patients with successful pET and defined WOI were selected for further in-depth analysis. To our knowledge, this is the first study with precise WOI dating by combining transcriptomic signatures and pregnancy outcomes in Chinese RIF patients. Overall, the clinical pregnancy rate in RIF patients has improved to 65% (26/40) after ERD-guided pET with adjusted WOI timing. The majority of RIF patients could achieve clinical pregnancy after adjusting the WOI according to ERD results, suggesting that asynchronous development of embryos and endometrium due to incorrect dating of WOI may be one of the main reasons for implantation failures. Combined together, these results in RIF patients have demonstrated the necessity of WOI dating before IVF-ET and suggested that WOI displacement may directly affect IVF-ET outcome and lead to RIF (12, 20).

In the current study, we performed the first endometrial transcriptome analysis of HRT cycle in Chinese RIF patients with defined WOI. Our transcriptome profiling identified significant differences in gene expression profiles between the P+5 endometrium from normal and displaced WOI groups. The biological processes and pathways enriched in advanced vs. normal WOI groups are similar to those of “late-receptive vs. receptive” (late-secretory vs. mid-secretory) reported before (47, 48). This indicates that the P+5 endometrium with advanced WOI is similar to the late-receptive phenotype with WOI tending to close. The biological processes enriched in delayed vs. normal WOI groups are similar to those of “pre-receptive vs. receptive” (pre-secretory vs. mid-secretory), suggesting that the P+5 endometrium with delayed WOI is not yet receptive and closer to the pre-receptive phenotype (44, 47). These data suggest that endometrium from advanced and delayed WOI groups were indeed altered and personalized WOI can be identified by transcriptome profiling.

As one of the main causes of RIF, WOI displacement and ER abnormality may be caused by some critical genes aberrantly expressed in the endometrium during WOI (18, 49–51). In current study, 10 genes were identified as novel candidates for distinguishing WOI displacement in RIF patients. *DPP4* encodes Dipeptidyl peptidase IV also known as T-cell activation antigen CD26, which participates in immune regulation, signal transduction, glucose homeostasis and apoptosis and plays an important role in successful pregnancy (33, 52). Both *CXCR1* and *CXCR2* encode the interleukin 8 (IL8) receptors that are highly expressed during WOI to enhance immune responses (53). The protein encoded by *LCN-2* (lipocalin 2) performs an essential role in the immune response by limiting bacterial growth and lcn2-deficient mice showed increased susceptibility to bacterial infection and a significant reduction in female fertility (54, 55). As a member of the IL-6 cytokine family, OSM participants in acute inflammatory reaction and cell growth (56, 57). These genes related to immunomodulation were supposed to increase their expression level in the secretory phase endometrium, but their expression levels were down-regulated 2~7-fold in P+5 endometrium with delayed WOI compared to normal endometrium. High *LRRC1* expression in mid-secretory endometrium may cause mutual repulsion between the embryo and endometrium and consequently lead to RIF (58). *TM4SF4* encodes member 4 protein of the transmembrane 4 superfamily which is involved in signal transduction, cell adhesion and regulation of ER (59). Additionally, *SLC25A48*, *CES4A* and *TNFRSF10C* are involved in the crucial processes of implantation, such as transmembrane transport, lipid metabolism and apoptosis (60). Together, these genes are deeply involved in ER functions and their aberrant expression in RIF patients may directly contribute to WOI displacement. Considering the key role of the 10 DEGs in identifying WOI displacement, these genes could be integrated into the gene set of the WOI prediction model when optimizing the ERD model.

Another important question is whether healthy fertile women in natural cycles and RIF patients in HRT cycles share similar endometrial gene expression patterns around WOI. Previously, the similar transcriptomic profile has been found during the transition of the endometrium from the pre-receptive to the receptive stage in both COS and natural cycles (61, 62). By combining our two studies on transcriptomic profiles of healthy and RIF women, we were able to assess the similarity of gene expression along WOI in detail. First, ERD developed on endometrial transcriptomic signatures from the natural cycle was able to distinguish the endometrium from the different phases of the HRT cycle and boosted IVF-ET outcome, suggesting transcriptomic similarities in both cycles. Global correlation analysis also suggested that the endometrial gene expression profiles of HRT cycles (P+3, P+5, and P+7) were largely similar to those of natural cycles (LH+5, LH+7, and LH+9). In addition, we found that genes with dynamic expression around WOI, including ER biomarkers such as *PAEP*, *SPP1*, and *C4BPA*, exhibited similar expression patterns in both cycles (28, 33, 44). Together, these results suggested that the human endometrium follows a common genetic program under natural and pathological conditions to achieve key biological functions.

Although new insights have been provided in RIF patients undergoing HRT cycles, the present study still has some limitations. First, these findings are currently validated only in the RIF patients recruited for this study. We have planned a 200-person-scale randomized controlled trial (RCT) to evaluate the clinical efficacy of the ERD test (also known as Tb-ERA) in Chinese RIF patients (38). Moreover, larger multicenter RCTs are required in the future to confirm these signatures in patients with different genetic backgrounds and/or different clinical complications. Second, both asynchronous (displaced) and pathological (destructed) WOIs exist in patients with RIF (20), but we excluded RIF patients with certain pathological complications in this study cohort. Third, even though gene expression profiles from both natural and HRT cycles showed similar and phase-specific patterns, unique signatures to RIF patients cannot be ruled out. Whether new ERD trained with transcriptome data from RIF patients can be beneficial deserves further investigation. We have planned to recruit more RIF patients for ERD testing in future studies to investigate the pathological factors causing receptive abnormalities and to further explore the transcriptomic profile of WOI under pathological conditions.

## 5 Conclusion

In summary, we suggest that the WOI displacements (ER changes during conventional WOI) in RIF patients are associated with abnormal expression of ER-related genes in the endometrium during HRT cycles. The 10 genes differentially expressed between the P+5 endometrium with normal or displaced WOIs identified in this study play essential roles in endometrial function and embryo implantation which can be used as biomarkers to recognize ER abnormalities and WOI displacement. Both natural and HRT cycles share similar transcriptome profiles during WOI. The improvement in the clinical pregnancy rate of RIF patients with ERD-guided pET suggests that ERD developed on data from natural cycles possess the potential for pWOI dating in RIF patients during HRT cycles. The clinical application of ERD could provide more precise timing of pET for RIF patients undergoing IVF-ET, and improve their pregnancy outcome.

## Data availability statement

The research data can be obtained by contacting the corresponding author. The 73 transcriptomic data from 26 RIF patients in this study are deposited in the Gene Expression Omnibus (GEO) database, accession number GSE252280.

## Ethics statement

The studies involving human participants were reviewed and approved by the ethical committee of Shanghai Ji Ai Genetics and IVF-ET Institute of Obstetrics and Gynecology hospital affiliated with Fudan University (JIAI E2020-015). The patients/participants provided their written informed consent to participate in this study.

## Author contributions

W-BZ and JL reviewed the literature, wrote the manuscript, and designed the figures and tables. XL, J-LC, LL, HC and WF participated in the methodology design, patient recruitment, samples collection and clinical validation. QL, J-CC and B-JL contributed to data curation, formal analysis, software and visualization of this work. HW and X-XS made substantial contributions to the conception and design of the work and provided input into manuscript content and composition. All authors contributed to the article and approved the submitted version.

## Supplementary material

Supplementary Table 1 Phase-grouped DEGs of endometrial samples from advanced, normal and delayed WOI groups.

Supplementary Table 2 DEGs identified by comparisons among the P+5 endometrial samples from advanced, normal and delayed WOI groups.

Supplementary Table 3 Clusters of the genes with similar expression patterns in natural and HRT cycles.

## References

[1] Achache H Revel A. Endometrial receptivity markers, the journey to successful embryo implantation. Hum Reprod Update (2006) 12(6):731–46. doi: 10.1093/humupd/dml004 16982667

[2] Kovalevsky G Patrizio P. High rates of embryo wastage with use of assisted reproductive technology: a look at the trends between 1995 and 2001 in the United States. Fertil Steril (2005) 84(2):325–30. doi: 10.1016/j.fertnstert.2005.04.020 16084872

[3] Smith A Tilling K Nelson SM Lawlor DA. Live-Birth rate associated with repeat *in vitro* fertilization treatment cycles. JAMA (2015) 314(24):2654–62. doi: 10.1001/jama.2015.17296 PMC4934614 26717030

[4] Ojosnegros S Seriola A Godeau AL Veiga A. Embryo implantation in the laboratory: an update on current techniques. Hum Reprod Update (2021) 27(3):501–30. doi: 10.1093/humupd/dmaa054 33410481

[5] Coughlan C Ledger W Wang Q Liu F Demirol A Gurgan T. Recurrent implantation failure: definition and management. Reprod BioMed Online (2014) 28(1):14–38. doi: 10.1016/j.rbmo.2013.08.011 24269084

[6] Macklon N. Recurrent implantation failure is a pathology with a specific transcriptomic signature. Fertil Steril (2017) 108(1):9–14. doi: 10.1016/j.fertnstert.2017.05.028 28602479

[7] Teh WT McBain J Rogers P. What is the contribution of embryo-endometrial asynchrony to implantation failure? J Assist Reprod Genet (2016) 33(11):1419–30. doi: 10.1007/s10815-016-0773-6 PMC5125144 27480540

[8] Young SL. Evaluation of endometrial function: a Heraclean or Sisyphean task? Fertil Steril (2017) 108(4):604–5. doi: 10.1016/j.fertnstert.2017.07.1166 PMC5683894 28965557

[9] Bellver J Simon C. Implantation failure of endometrial origin: what is new? Curr Opin Obstet Gynecol (2018) 30(4):229–36. doi: 10.1097/GCO.0000000000000468 29889670

[10] Kliman HJ Frankfurter D. Clinical approach to recurrent implantation failure: evidence-based evaluation of the endometrium. Fertil Steril (2019) 111(4):618–28. doi: 10.1016/j.fertnstert.2019.02.011 30929719

[11] Koot YE van Hooff SR Boomsma CM van Leenen D Groot Koerkamp MJ Goddijn M. An endometrial gene expression signature accurately predicts recurrent implantation failure after IVF. Sci Rep (2016) 6:19411. doi: 10.1038/srep19411 26797113 PMC4726345

[12] Ruiz-Alonso M Blesa D Diaz-Gimeno P Gomez E Fernandez-Sanchez M Carranza F. The endometrial receptivity array for diagnosis and personalized embryo transfer as a treatment for patients with repeated implantation failure. Fertil Steril (2013) 100(3):818–24. doi: 10.1016/j.fertnstert.2013.05.004 23756099

[13] Hashimoto T Koizumi M Doshida M Toya M Sagara E Oka N. Efficacy of the endometrial receptivity array for repeated implantation failure in Japan: A retrospective, two-centers study. Reprod Med Biol (2017) 16(3):290–6. doi: 10.1002/rmb2.12041 PMC5715887 29259480

[14] Harper MJ. The implantation window. Baillieres Clin Obstet Gynaecol (1992) 6(2):351–71. doi: 10.1016/S0950-3552(05)80092-6 1424330

[15] Gómez E Ruíz-Alonso M Miravet J Simón C. Human endometrial transcriptomics: implications for embryonic implantation. Cold Spring Harbor Perspect Med (2015) 5(7). doi: 10.1101/cshperspect.a022996 PMC4484960 25818663

[16] Mumusoglu S Polat M Ozbek IY Bozdag G Papanikolaou EG Esteves SC. Preparation of the endometrium for frozen embryo transfer: A systematic review. Front Endocrinol (2021) 12. doi: 10.3389/fendo.2021.688237 PMC8299049 34305815

[17] Galliano D Bellver J Diaz-Garcia C Simon C Pellicer A. ART and uterine pathology: how relevant is the maternal side for implantation? Hum Reprod Update (2015) 21(1):13–38. doi: 10.1093/humupd/dmu047 25155826

[18] Messaoudi S El Kasmi I Bourdiec A Crespo K Bissonnette L Le Saint C. 15 years of transcriptomic analysis on endometrial receptivity: what have we learnt? Fertil Res Pract (2019) 5:9. doi: 10.1186/s40738-019-0059-7 31396393 PMC6681490

[19] Franasiak JM Ruiz-Alonso M Scott RT Simon C. Both slowly developing embryos and a variable pace of luteal endometrial progression may conspire to prevent normal birth in spite of a capable embryo. Fertil Steril (2016) 105(4):861–6. doi: 10.1016/j.fertnstert.2016.02.030 26940791

[20] Sebastian-Leon P Garrido N Remohi J Pellicer A Diaz-Gimeno P. Asynchronous and pathological windows of implantation: two causes of recurrent implantation failure. Hum Reprod (2018) 33(4):626–35. doi: 10.1093/humrep/dey023 29452422

[21] Tan J Kan A Hitkari J Taylor B Tallon N Warraich G. The role of the endometrial receptivity array (ERA) in patients who have failed euploid embryo transfers. J Assist Reprod Genet (2018) 35(4):683–92. doi: 10.1007/s10815-017-1112-2 PMC5949105 29327111

[22] Haouzi D Entezami F Torre A Innocenti C Antoine Y Mauries C. Customized frozen embryo transfer after identification of the receptivity window with a transcriptomic approach improves the implantation and live birth rates in patients with repeated implantation failure. Reprod Sci (2021) 28(1):69–78. doi: 10.1007/s43032-020-00252-0 32725589 PMC7782404

[23] Craciunas L Gallos I Chu J Bourne T Quenby S Brosens JJ. Conventional and modern markers of endometrial receptivity: a systematic review and meta-analysis. Hum Reprod Update (2019) 25(2):202–23. doi: 10.1093/humupd/dmy044 30624659

[24] Kang YJ Forbes K Carver J Aplin JD. The role of the osteopontin-integrin alphavbeta3 interaction at implantation: functional analysis using three different *in vitro models* . Hum Reprod (2014) 29(4):739–49. doi: 10.1093/humrep/det433 24442579

[25] Serafini P Rocha AM Osorio CT da Silva I Motta EL Baracat EC. Endometrial leukemia inhibitory factor as a predictor of pregnancy after *in vitro* fertilization. Int J Gynaecol Obstet (2008) 102(1):23–7. doi: 10.1016/j.ijgo.2007.12.005 18289544

[26] Taylor HS Arici A Olive D Igarashi P. HOXA10 is expressed in response to sex steroids at the time of implantation in the human endometrium. J Clin Invest (1998) 101(7):1379–84. doi: 10.1172/JCI1597 PMC508715 9525980

[27] Domínguez F Muñoz M Hernández-Vargas P. Identifying biomarkers for predicting successful embryo implantation: applying single to multi-OMICs to improve reproductive outcomes. Hum Reprod Update (2020) 26(2):264–301. doi: 10.1093/humupd/dmz042 32096829

[28] Diaz-Gimeno P Horcajadas JA Martinez-Conejero JA Esteban FJ Alama P Pellicer A. A genomic diagnostic tool for human endometrial receptivity based on the transcriptomic signature. Fertil Steril (2011) 95(1):50–60. doi: 10.1016/j.fertnstert.2010.04.063 20619403

[29] Garrido-Gomez T Ruiz-Alonso M Blesa D Diaz-Gimeno P Vilella F Simon C. Profiling the gene signature of endometrial receptivity: clinical results. Fertil Steril (2013) 99(4):1078–85. doi: 10.1016/j.fertnstert.2012.12.005 23312228

[30] Simon C Gomez C Cabanillas S Vladimirov I Castillon G Giles J. A 5-year multicentre randomized controlled trial comparing personalized, frozen and fresh blastocyst transfer in IVF. Reprod BioMed Online (2020) 41(3):402–15. doi: 10.1016/j.rbmo.2020.06.002 32723696

[31] McGettigan PA. Transcriptomics in the RNA-seq era. Curr Opin Chem Biol (2013) 17(1):4–11. doi: 10.1016/j.cbpa.2012.12.008 23290152

[32] Ben Rafael Z. Endometrial Receptivity Analysis (ERA) test: an unproven technology. Hum Reprod Open (2021) 2021(2):hoab010. doi: 10.1093/hropen/hoab010 33880419 PMC8045470

[33] Altmäe S Koel M Võsa U Adler P Suhorutšenko M Laisk-Podar T. Meta-signature of human endometrial receptivity: a meta-analysis and validation study of transcriptomic biomarkers. Sci Rep (2017) 7(1):10077. doi: 10.1038/s41598-017-10098-3 28855728 PMC5577343

[34] Haouzi D Mahmoud K Fourar M Bendhaou K Dechaud H De Vos J. Identification of new biomarkers of human endometrial receptivity in the natural cycle. Hum Reprod (2008) 24(1):198–205. doi: 10.1093/humrep/den360 18835874

[35] Riesewijk A Martin J van Os R Horcajadas JA Polman J Pellicer A. Gene expression profiling of human endometrial receptivity on days LH+2 versus LH+7 by microarray technology. Mol Hum Reprod (2003) 9(5):253–64. doi: 10.1093/molehr/gag037 12728018

[36] Zhang WB Li Q Liu H Chen WJ Zhang CL Li H. Transcriptomic analysis of endometrial receptivity for a genomic diagnostics model of Chinese women. Fertil Steril (2021) 116(1):157–64. doi: 10.1016/j.fertnstert.2020.11.010 33589135

[37] Balaban B Brison D Calderon G Catt J Conaghan J Cowan L. The Istanbul consensus workshop on embryo assessment: proceedings of an expert meeting. Hum Reprod (2011) 26(6):1270–83. doi: 10.1093/humrep/der037 21502182

[38] Zhang WB Li H Lu X Chen JL Li L Chen JC. The clinical efficiency of transcriptome-based endometrial receptivity assessment (Tb-ERA) in Chinese patients with recurrent implantation failure (RIF): A study protocol for a prospective randomized controlled trial. Contemp Clin Trials Commun (2022) 28:100928. doi: 10.1016/j.conctc.2022.100928 35669489 PMC9163422

[39] Chen S Zhou Y Chen Y Gu J. fastp: an ultra-fast all-in-one FASTQ preprocessor. Bioinformatics (2018) 34(17):i884–90. doi: 10.1093/bioinformatics/bty560 PMC6129281 30423086

[40] Dobin A Davis CA Schlesinger F Drenkow J Zaleski C Jha S. STAR: ultrafast universal RNA-seq aligner. Bioinformatics (2013) 29(1):15–21. doi: 10.1093/bioinformatics/bts635 23104886 PMC3530905

[41] Kopylova E Noe L Touzet H. SortMeRNA: fast and accurate filtering of ribosomal RNAs in metatranscriptomic data. Bioinformatics (2012) 28(24):3211–7. doi: 10.1093/bioinformatics/bts611 23071270

[42] Wagner GP Kin K Lynch VJ. Measurement of mRNA abundance using RNA-seq data: RPKM measure is inconsistent among samples. Theory Biosci (2012) 131(4):281–5. doi: 10.1007/s12064-012-0162-3 22872506

[43] Harrow J Frankish A Gonzalez JM Tapanari E Diekhans M Kokocinski F. GENCODE: the reference human genome annotation for The ENCODE Project. Genome Res (2012) 22(9):1760–74. doi: 10.1101/gr.135350.111 PMC3431492 22955987

[44] Ruiz-Alonso M Blesa D Simon C. The genomics of the human endometrium. Biochim Biophys Acta (2012) 1822(12):1931–42. doi: 10.1016/j.bbadis.2012.05.004 22634130

[45] Enciso M Carrascosa JP Sarasa J Martinez-Ortiz PA Munne S Horcajadas JA. Development of a new comprehensive and reliable endometrial receptivity map (ER Map/ER Grade) based on RT-qPCR gene expression analysis. Hum Reprod (2018) 33(2):220–8. doi: 10.1093/humrep/dex370 29315421

[46] Diaz-Gimeno P Ruiz-Alonso M Blesa D Bosch N Martinez-Conejero JA Alama P. The accuracy and reproducibility of the endometrial receptivity array is superior to histology as a diagnostic method for endometrial receptivity. Fertil Steril (2013) 99(2):508–17. doi: 10.1016/j.fertnstert.2012.09.046 23102856

[47] Talbi S Hamilton AE Vo KC Tulac S Overgaard MT Dosiou C. Molecular phenotyping of human endometrium distinguishes menstrual cycle phases and underlying biological processes in normo-ovulatory women. Endocrinology (2006) 147(3):1097–121. doi: 10.1210/en.2005-1076 16306079

[48] Tseng LH Chen I Chen MY Yan H Wang CN Lee CL. Genome-based expression profiling as a single standardized microarray platform for the diagnosis of endometrial disorder: an array of 126-gene model. Fertil Steril (2010) 94(1):114–9. doi: 10.1016/j.fertnstert.2009.01.130 19328470

[49] Fan LJ Han HJ Guan J Zhang XW Cui QH Shen H. Aberrantly expressed long noncoding RNAs in recurrent implantation failure: A microarray related study. Syst Biol Reprod Med (2017) 63(4):269–78. doi: 10.1080/19396368.2017.1310329 28441042

[50] Koler M Achache H Tsafrir A Smith Y Revel A Reich R. Disrupted gene pattern in patients with repeated *in vitro* fertilization (IVF) failure. Hum Reprod (2009) 24(10):2541–8. doi: 10.1093/humrep/dep193 19542175

[51] Tapia A Gangi LM Zegers-Hochschild F Balmaceda J Pommer R Trejo L. Differences in the endometrial transcript profile during the receptive period between women who were refractory to implantation and those who achieved pregnancy. Hum Reprod (2008) 23(2):340–51. doi: 10.1093/humrep/dem319 18077318

[52] Horcajadas JA Pellicer A Simon C. Wide genomic analysis of human endometrial receptivity: new times, new opportunities. Hum Reprod Update (2007) 13(1):77–86. doi: 10.1093/humupd/dml046 16960016

[53] Mulayim N Palter SF Kayisli UA Senturk L Arici A. Chemokine receptor expression in human endometrium. Biol Reprod (2003) 68(5):1491–5. doi: 10.1095/biolreprod.102.009639 12606476

[54] Berger T Togawa A Duncan GS Elia AJ You-Ten A Wakeham A. Lipocalin 2-deficient mice exhibit increased sensitivity to Escherichia coli infection but not to ischemia-reperfusion injury. Proc Natl Acad Sci USA (2006) 103(6):1834–9. doi: 10.1073/pnas.0510847103 PMC1413671 16446425

[55] Grutzner F Rens W Tsend-Ayush E El-Mogharbel N O'Brien PC Jones RC. In the platypus a meiotic chain of ten sex chromosomes shares genes with the bird Z and mammal X chromosomes. Nature (2004) 432(7019):913–7. doi: 10.1038/nature03021 15502814

[56] Modur V Feldhaus MJ Weyrich AS Jicha DL Prescott SM Zimmerman GA. Oncostatin M is a proinflammatory mediator. *In vivo* effects correlate with endothelial cell expression of inflammatory cytokines and adhesion molecules. J Clin Invest (1997) 100(1):158–68. doi: 10.1172/JCI119508 PMC508176 9202068

[57] Rose TM Bruce AG. Oncostatin M is a member of a cytokine family that includes leukemia-inhibitory factor, granulocyte colony-stimulating factor, and interleukin 6. Proc Natl Acad Sci USA (1991) 88(19):8641–5. doi: 10.1073/pnas.88.19.8641 PMC52565 1717982

[58] Huang J Song N Xia L Tian L Tan J Chen Q. Construction of lncRNA-related competing endogenous RNA network and identification of hub genes in recurrent implantation failure. Reprod Biol Endocrinol (2021) 19(1):108. doi: 10.1186/s12958-021-00778-1 34243770 PMC8268333

[59] Qiao J Wang L Li R Zhang X. Microarray evaluation of endometrial receptivity in Chinese women with polycystic ovary syndrome. Reprod BioMed Online (2008) 17(3):425–35. doi: 10.1016/S1472-6483(10)60228-3 18765015

[60] Palmieri F. The mitochondrial transporter family SLC25: identification, properties and physiopathology. Mol Aspects Med (2013) 34(2-3):465–84. doi: 10.1016/j.mam.2012.05.005 23266187

[61] Horcajadas JA Minguez P Dopazo J Esteban FJ Dominguez F Giudice LC. Controlled ovarian stimulation induces a functional genomic delay of the endometrium with potential clinical implications. J Clin Endocrinol Metab (2008) 93(11):4500–10. doi: 10.1210/jc.2008-0588 18697870

[62] Haouzi D Dechaud H Assou S De Vos J Hamamah S. Insights into human endometrial receptivity from transcriptomic and proteomic data. Reprod BioMed Online (2012) 24(1):23–34. doi: 10.1016/j.rbmo.2011.09.009 22119322

